# School Engagement and Context: A Multilevel Analysis of Adolescents in 31 Provincial-Level Regions in China

**DOI:** 10.3389/fpsyg.2021.724819

**Published:** 2021-10-26

**Authors:** Fangqing Liu, Xiaosong Gai, Lili Xu, Xiaojing Wu, Hong Wang

**Affiliations:** School of Psychology, Northeast Normal University, Changchun, China

**Keywords:** adolescents, school engagement, SES, provincial environment, ecological system theory

## Abstract

According to ecological system theory, both the microsystem environment (home environment) and the more macrolevel environment (provincial environment) influence school engagement in adolescents. This study tests an ecological model of adolescents’ school engagement with 19,084 middle school students across 31 provincial-level regions in China. Multilevel modeling is used to predict adolescents’ school engagement (behavior, emotion, and cognition) at two levels, individual [gender and family socioeconomic status (SES)] and provincial (economy, public cultural facilities, technological industry and education). The school engagement of students varies significantly across provincial-level regions. SES positively affects the school engagement of students. Students benefit from the provincial environment when the economy is booming, public cultural facilities are adequate and education is flourishing. The development of the technology industry fails to boost students’ school engagement. Limitations and future directions are discussed.

## Introduction

School engagement, which is a way to predict academic performance and student boredom, has become an increasingly important concept for education researchers (Wang and Fredricks, 2014). Fredricks et al. (2004) define school engagement in three ways. *Behavioral engagement* refers to participation in learning activities; it includes involvement in academic activities and extracurricular activities. It is considered crucial for positive academic achievement and preventing dropping out. *Emotional engagement* refers to students’ emotional response in learning activities, including positive or negative reactions to teachers, classmates, academics and school. It is considered a tie linking an institution and willingness to do the work. *Cognitive engagement* refers to the willingness and effort to understand complex ideas and master difficult skills, including the use of learning strategies and self-regulating learning methods and the use of metacognitive strategies to plan and evaluate learning.

Many studies have indicated that school engagement has a close relation to students’ academic achievement and academic adjustment (Lam et al., 2012; Chase et al., 2014; Galla et al., 2014; Stefansson et al., 2018; Zhu et al., 2019; Xiong et al., 2021; Zhao et al., 2021). Some researchers consider school engagement a predictor of the quality of education (Reina et al., 2014; Li, 2018). It has been proven that school engagement is a way to ameliorate academic burnout (Duan and Li, 2008; Robayo-Tamayo et al., 2020) and dropout (Janosz et al., 2008; Wang and Fredricks, 2014).

In recent years, two gaps in the school engagement literature have been identified. First, there is a need to examine multiple layers of environmental factors regarding their role in enhancing or undermining adolescents’ school engagement (Mohammadpour, 2013; Camacho and Krezmien, 2018). According to Bronfenbrenner (1989), human development occurs in a nested environmental system. Human development is directly or indirectly affected by four interacting systems (Bronfenbrenner and Morris, 2006). Microsystems are the most proximal and influential systems impacting adolescents’ developmental outcomes. Adolescents continually interact with others and carry out daily activities in microsystems. The bidirectional interactions between adolescents and the microsystem context directly influence adolescents’ development (Kim, 2015). Prior studies have examined individual factors (age) and microsystem factors (home environment, family environment, parenting, neighborhood context, teacher quality, and school context) that predict students’ school engagement (Chiu et al., 2012; Lam et al., 2012; Wang and Eccles, 2012a; Shi et al., 2013; Wang and Chang, 2018; Harris et al., 2020). The macrosystem is the most distal and broadest context and influences adolescents’ development and other contexts (including microsystems). Education outcomes and the economy, politics, culture, technology and other societal factors are interdependent and mutually restricted (Feng, 2007). A limited number of studies provide proof that school engagement differs between macrosystem environments (Lam et al., 2015; Nguyen et al., 2018) and is influenced by gross domestic product (GDP) and income inequality (GINI) (Dotterer and Lowe, 2011). Second, few studies have provided robust proof of the interaction of microsystems and macrosystems and how the interaction influences adolescents’ school engagement. According to ecosystem theory, the environment influences each other. Some questions remained unclear. How did they interact with each other? Whether they compensate each other? To simplify sampling and out of consideration for research costs, most studies focus on the influence of microsystem factors on school engagement in adolescents, and only a few studies report findings about macrosystem effects. Therefore, it is difficult to draw a reliable conclusion regarding the interaction of microsystems and macrosystems.

### Adolescent-Level Individual Factor: Gender

Historically, gender has been a predictor of school engagement. Several studies have investigated gender differences in school engagement. Some studies report that girls have higher school engagement than boys (Freudenthaler et al., 2008; Janosz et al., 2008; Wang and Eccles, 2012a; Wang and Fredricks, 2014). Other studies, however, have found contradictory or even no effects of gender on adolescents’ school engagement (Ruban and McCoach, 2005; Janosz et al., 2008; Steinmayr and Spinath, 2008; Wilcox et al., 2017; Zendarski et al., 2017). Participants in the studies were from different groups in different regions, which may account for the inconsistent results. For example, Wang and Fredricks (2014) recruited 7th students from an ethnically diverse county on the East Coast of the United States. Janosz et al. (2008) recruited 7th to 11th students from low socioeconomic (SES) middle and high schools in Canada.

### Adolescent-Level Microsystem Factor: Family Socioeconomic Status

According to ecological system theory (Bronfenbrenner and Ceci, 1994), microsystems are the most proximal and influential systems impacting children’s developmental outcomes. Adolescents continually interact with others and carry out daily activities in microsystems. The bidirectional interactions between adolescents and the microsystem context directly influence adolescents’ development. Microsystems, such as family context, have typically been proven to influence adolescents’ academic activities (Sağkal, 2019).

The associations between student school engagement and the home microsystem have been examined extensively. Some studies have reported consistent results that students from higher SES homes exhibit more engagement in school work than those from lower SES homes (Ruban and McCoach, 2005; Janosz et al., 2008; Ni and Wu, 2011; Wang and Eccles, 2012b; Akiva et al., 2013; Shi et al., 2013; Wang and Chang, 2018). Adolescents from high-SES families have more access to resources (e.g., extracurricular activities and books) and less pressure. Adolescents from low-SES families have less access to resources and often have to take on additional responsibilities and pressures because of working parents (Duan et al., 2018).

### Province-Level Macrosystem Factors

According to Bronfenbrenner (1994), the macrosystem is the most distal context in adolescents’ environment, but it can have imperceptible effects on other contexts (including microsystems) and adolescents’ development. The macrosystem includes the economy, politics, culture, technology, education, and other aspects of the environment in which adolescents grow up. A limited number of studies have provided evidence for the impact of macrosystems on adolescent school engagement. Lam et al. (2015) found that the school engagement of students was significantly different across 12 countries. They examined how regional differences in the environment affect adolescents’ school engagement. Two other studies found that GDP and the GINI coefficient can significantly affect reading performance (Dotterer and Lowe, 2011; Chiu et al., 2017). According to the significant relationship between school engagement and academic achievement (Kasehagen et al., 2018; Xiong et al., 2021), GDP and the GINI coefficient were potential factors affecting school engagement.

The relationship between the microsystem environment (family, class, and school) and the development of youth has been examined widely (Dotterer and Lowe, 2011; Vanwynsberghe et al., 2017; Harris et al., 2020). However, few studies have focused on the association of macrosystems (such as the urban environment) with the academic development of adolescents. Although effects of the economic and wealth gap on the academic performance of adolescents have been found, the impact of other environmental factors on adolescent development is unclear. Moreover, the interaction between the macrosystem and microsystem needs to be examined (Lam et al., 2015).

This study obtained the school engagement scores and family SES of middle school students from 31 provincial regions in China and collected the economic, public cultural service, scientific and technological development and education indicators of the 31 provincial-level regions. A multilevel analysis model was used to explore the influence of SES and provincial index on the school engagement of middle school students and the interaction between the province environment and family environment. The following research questions were used to guide this study:

Question 1. Did individual factors (gender and SES) influence adolescents’ school engagement?

Question 2. What was the influence of provincial-level factors on adolescents’ school engagement?

Question 3. Was there an interaction between individual factors and provincial-level factors on adolescents’ school engagement?

## Materials and Methods

### Participants

Participants for the current study were from the Adolescent Purpose of Life Project, which was designed to test the relationship between adolescents’ purpose and development outcome. We recruited schools with cooperation intentions on the website. We promised to provide the analysis and report of the current development of their students and lectures on mental health for free. We recruited 183 middle schools in 31 provincial-level regions in China. The provincial-level regions include 22 provinces (all provinces except Taiwan), 5 autonomous regions and 4 municipalities. Teachers of partner school recruited students and obtained the consent of the students and their parents.

Two methods were used to collect the data. First, teachers posted the questionnaire to a website, and the students responded online. Second, teachers distributed a paper questionnaire, and students wrote their answers. All the data were collected in August 2019. The original sample consisted of 22,469 adolescents. The data was preprocessed in three steps. First, we selected participants with high social expectations. Five questions were used to test social expectations. They were “I never cry,” “I never break an appointment,” “I never swear,” “I never eat snacks” and “I never lie.” The response formats of the items ranged from 1 = strongly disagree to 5 = strongly agree. We removed the participants who scored more than four points on these five items. We removed 2,982 participants at this step. Second, we selected the participants with a blank question rate of more than 15%. We removed 212 participants at this step. Third, we removed the data with extreme values. Some data were collected as a pencil and paper study. Later, the study found extreme values above the maximum values of the Likert scale (4), because of a mistake in data entry. We removed 91 participants at this step. The final sample consisted of 19,084 adolescents (boys = 9,593 and girls = 9,421; Mage = 13.96; *SD* = 0.82). Detailed information on the participant distribution is provided in Appendix.

### Measures

#### School Engagement Questionnaire

School engagement was measured using the School Engagement Questionnaire (Liu et al., Submitted^1^). This questionnaire consisted of three subscales: behavioral, emotional and cognitive. The behavioral engagement subscale consisted of 5 items that measure adolescents’ effort in learning (e.g., “I work hard at school”). The cognitive engagement subscale consisted of 7 items that measure the use of metacognition strategies in learning (e.g., “I always check my homework”). The emotional engagement subscale consisted of 6 items that measure adolescents’ feelings of learning and school (e.g., “I feel boring in class”). The questionnaire was based on a 4-point Likert-type scale (strongly disagree to strongly agree). Cronbach’s alphas were 0.85 (cognitive), 0.85 (emotional) and 0.82 (behavioral) in the current study. Confirmatory factor analysis showed that the factor load of all items ranged from 0.58 to 0.87, and the model had good fit, as shown in Table 1.

**TABLE 1:** School engagement model fitting index.

χ^2^	*df*	χ^2^*/df*	RMSEA	CFI	TLI	SRMR
387.09	130	2.98	0.06	0.94	0.93	0.04

#### Family Socioeconomic Status

Parents’ occupation and education level together represent SES in the current study (Bradley and Corwyn, 2002). According to the occupational classification (Lin and Bian, 1991; Shi and Shen, 2007), we classified occupations into nine categories as in the following example: In terms of occupation, is your father (if not one of the following, please choose the most relevant option) unemployed, a service worker or manual laborer (e.g., farmer or waiter), a transactional worker (e.g., secretary, clerk), self-employed with no or a few employees, the owner of a large or medium-sized enterprise, a corporate middle manager, military or police personnel, professional or technical personnel (e.g., doctor, designer, teacher, engineer, accountant, lawyer), or a national public official (e.g., civil servant)? Is your father’s education level elementary school and junior middle school, high school or technical secondary school, college, university, or graduate and above? The first four parental occupation categories were coded 1, and the last five were coded 2; the education degree was coded 1, 2, 3, 4, 5, or 6. SES was calculated by adding education level and occupation of parents Z-transformed scores together.

Province-level variables. Six indicators reflecting the provincial environment in terms of the economy, culture, education, and science and technology were constructed with data from the National Bureau of Statistics and the Ministry of Education.

#### Economy

We used real GDP per capita (GDP_pc_) in 2019 as an indicator of the state of the economy. This index is calculated as the province’s GDP divided by the province’s total population.

#### Public Cultural Service System

We used the number of public library books per capita (PB_pc_) in 2011 as an indicator of culture. This index is calculated as the total number of books held by public libraries in the province divided by the total number of people in the province.

#### Education

Three indicators were used to indicate the development of education: percentage of the population who had received higher education (PHE), educational appropriations per student (EA_ps_) and student-teacher ratio (STR). PHE, EA_ps_ and STR were collected in 2019.

#### Scientific and Technological Development

The per capita technology market transaction amount (TMTA_pc_) in 2019 was used as the science and technology indicator. This index is calculated as the total transaction number of registered contracts in the province divided by the total number of people in the province.

### Model-Building Approach

We ran a series of two-level hierarchical linear models (HLMs) using mixed models in SPSS 23 to address all the research questions. The model-building approach began with an intercept-only model to serve as a baseline and provide the intraclass correlation (ICC). Subsequently, the level-1 predictors (SES and gender), the level-2 predictors (i.e., GDP_pc_, PB_pc_, PHE, EA_ps_, STR, TMTA_pc_), and finally, the predictors of slopes (i.e., the slope of SES regressed on GDP_pc_) were added. We did not perform centering in our multilevel analyses.

The model was built by adding gender (male = 0, female = 1) and SES as adolescent-level predictors and GDP_pc_, PB_pc_, PHE, EA_ps_, STR, and TMTA_pc_ as province-level predictors. Considering the significant correlation between provincial indicators, we analyzed provincial indicators one by one in order to reduce the spurious correlation caused by collinearity. The correlation of provincial indicators was in Supplementary Material.

Level 1 (adolescent): *Y_ij_* = β*_*oi*_* + β*_1i_X_*ij*_* + β*_1i_ S_*ij*_* + ε*_*ij*_*

Level 2 (province): β*oi* = γ*_00_* + γ*_01_W_1i_* + μ*_*oi*_*

β*_1i_* = γ*_10_* + γ*_1i_W_1i_* + μ*_1i_*

The school engagement score of adolescent *i* in province *j* (*Y*_*ij*_) was modeled as a function of the mean school engagement score for province j (βoi). *X*_*ij*_ is the gender of adolescent i in province *j*. *S*_*ij*_ is the SES of adolescent i in province *j. W*_1i_ represents the level-2 variables, and X is a vector of the province variables. ε*_*ij*_* and μoi are residual terms signaling individual adolescent and province differences.

## Results

We specified a null parameter that is used to calculate the ICC, which estimates how much variation in school engagement exists between level-2 (province-level) units. The ICCs related to behavioral, emotional, and cognitive engagement, *τ00/*(*τ00* + σ*2*), were 0.06, 0.06, and 0.05, respectively. According to Peugh (2010), ICC values ranging from.05 to.20 are *common* in cross-sectional multilevel modeling studies. Thus, multilevel modeling was suitable for this study. Behavioral, emotional, and cognitive engagement varied significantly across provincial-level regions (β = 2.98, *t* = 105.25, *p* = 0.00, 95% *CI* = 2.92∼3.04; β = 3.04, *t* = 106.21, *p* = 0.00, 95% *CI* = 2.98∼3.10; β = 2.91, *t* = 116.33, *p* = 0.00, 95% *CI* = 2.86∼2.97) (see Tables 2–4). SES significantly predicted behavioral, emotional, and cognitive engagement (β = 0.11, *t* = 19.29, *p* = 0.00, 95% *CI* = 0.10∼0.13, *R*^2^*_*SES*_* = 1.78%; β = 0.10, *t* = 8.47, *p* = 0.00, 95% *CI* = 0.08∼0.13, *R*^2^*_*SES*_* = 1.57%; β = 0.11, *t* = 9.31, *p* = 0.00, 95% *CI* = 0.08∼0.13, *R*^2^*_*SES*_* = 1.77%). Gender failed to predict behavioral, emotional, and cognitive engagement (β = 0.01, *t* = 0.50, *p* = 0.62; β = 0.03, *t* = 2.13, *p* = 0.60; β = −0.02, *t* = −1.56, *p* = 0.18).

**TABLE 2:** The effect of gender, SES and provincial factors on behavioral engagement.

	Model 1		Model 2		Model 3		Model 4	
	β	*t*	β	*t*	β	*t*	β	*t*
Intercept	2.98	105.25***	2.97	127.36***	2.89	64.89***		
SES			0.12	9.62***	0.10	9.85***		
Gender			0.01	0.50	0.02	1.28		
GDP_pc_					0.00000002	1.73		
*R* ^2^		0.06		0.04		0.05		
Intercept	2.98	105.25***	2.97	127.36***	2.88	78.15***	2.87	77.96***
SES			0.12	9.62***	0.10	9.92***	0.10	4.91***
Gender			0.01	0.50	0.02	1.32	0.02	1.32
PB_pc_					0.11	2.72**	0.11	2.71**
SES* PB_pc_							−0.002	–0.12
R^2^		0.06		0.04		0.05		0.05
Intercept	2.98	105.25***	2.97	127.36***	2.83	63.60***	2.84	63.52***
SES			0.12	9.62***	0.10	9.78***	0.11	4.78***
Gender			0.01	0.50	0.02	1.30	0.02	1.29
PHE					0.11	3.04***	0.11	2.99***
SES* PHE							−0.01	–0.53
*R* ^2^		0.06		0.04		0.04		0.04
Intercept	2.98	105.25***	2.97	127.36***	2.84	64.59***	2.85	64.61***
SES			0.12	9.62***	0.10	9.77***	0.11	5.20***
Gender			0.01	0.50	0.02	1.3	0.02	1.29
EA_ps_					0.00000008	2.88***	0.000006	2.80**
SES* EA_ps_							−0.0000007	–0.72
*R* ^2^		0.06		0.04		0.05		0.05
Intercept	2.98	105.25***	2.97	127.36***	3.45	31.08***	3.47	30.66***
SES			0.12	9.62***	0.10	10.15***	0.16	2.46*
Gender			0.01	0.50	0.02	1.34	0.02	1.39
STR					−0.04	−4.50***	−0.04	−4.54***
SES* STR							−0.00	–0.72
*R* ^2^		0.06		0.04		0.04		0.04
Intercept	2.98	105.25***	2.97	127.36***	2.94	147.23***		
SES			0.12	9.62***	0.10	9.90***		
Gender			0.01	0.50	0.02	1.28		
TMTA_pc_					0.09	1.71		
*R* ^2^		0.06		0.04		0.05		

***p < 0.001, **p < 0.01, *p < 0.05.

**TABLE 3:** The effect of gender, SES and provincial factors on emotional engagement.

	Model 1		Model 2		Model 3		Model 4	
	β	*t*	β	*t*	β	*t*	β	*t*
Intercept	3.04	106.21***	3.02	118.68***	2.93	61.43***		
SES			0.11	8.91***	0.08	8.15***		
Gender			0.03	2.13	0.04	2.87**		
GDP_pc_					0.000001	1.75		
*R* ^2^		0.06		0.06		0.06		
Intercept	3.04	106.21***	3.02	118.68***	2.93	72.44***	2.92	71.70***
SES			0.11	8.91***	0.08	8.23***	0.07	3.52***
Gender			0.03	2.13	0.04	2.88**	0.04	2.88**
PB_pc_					0.10	2.24*	0.10	2.26*
SES* PB_pc_							0.01	0.47
*R* ^2^		0.06		0.06		0.06		0.06
Intercept	3.04	106.21***	3.02	118.68***	2.92	58.48***		
SES			0.11	8.91***	0.08	8.16***		
Gender			0.03	2.13	0.04	2.88***		
PHE					0.07	1.77		
*R* ^2^		0.06		0.06		0.06		
Intercept	3.04	106.21***	3.02	118.68***	2.93	60.23***		
SES			0.11	8.91***	0.08	8.23***		
Gender			0.03	2.13	0.04	2.89**		
EA_ps_					0.0001	1.69		
*R* ^2^		0.06		0.06		0.06		
Intercept	3.04	106.21***	3.02	118.68***	3.40	26.77***	3.42	26.13***
SES			0.11	8.91***	0.09	8.26***	0.15	2.14*
Gender			0.03	2.13	0.04	2.83**	0.04	2.85**
STR					-0.03	−3.14**	−0.03	−3.23***
SES* STR							−0.01	–0.93
*R* ^2^		0.06		0.06		0.06		0.06
Intercept	3.04	106.21***	3.02	118.68***	3.00	130.31***		
SES			0.11	8.91***	0.08	8.23***		
Gender			0.03	2.13	0.04	2.89**		
TMTA_pc_					0.04	0.66		
*R* ^2^		0.06		0.06		0.06		

***p < 0.001, **p < 0.01, *p < 0.05.

**TABLE 4:** The effect of gender, SES and provincial factors on cognition engagement.

	Model 1		Model 2		Model 3		Model 4	
	β	*t*	β	*t*	β	*t*	β	*t*
Intercept	2.91	116.33***	2.92	155.07***	2.83	75.48***	2.83	75.33***
SES			0.11	8.77***	0.09	10.42***	0.10	4.59
Gender			−0.02	–1.56	−0.01	–0.73	−0.01	–0.74
GDP_pc_					0.000001	2.12*	0.000001	2.10*
SES* GDP_pc_							−0.00000006	–0.22
*R* ^2^		0.05		0.05		0.03		0.03
Intercept	2.91	116.33***	2.92	155.07***	2.81	93.19***	2.81	92.62***
SES			0.11	8.77***	0.09	10.66***	0.09	4.76***
Gender			−0.02	–1.56	−0.01	–0.64	−0.01	–0.63
PB_pc_					0.11	3.53***	0.11	3.47***
SES* PB_pc_							0.01	0.31
*R* ^2^		0.05		0.05		0.03		0.03
Intercept	2.91	116.33***	2.92	155.07***	2.80	74.74***	2.80	74.40***
SES			0.11	8.77***	0.09	10.41***	0.09	10.41***
Gender			−0.02	–1.56	−0.01	–0.68	−0.01	–0.68
PHE					0.09	3.10***	0.09	3.10***
SES* PHE							0.0004	0.03
*R* ^2^		0.05		0.05		0.03		0.03
Intercept	2.91	116.33***	2.92	155.07***	2.81	75.99***	2.81	75.92***
SES			0.11	8.77***	0.09	10.36***	0.10	5.01***
Gender			−0.02	–1.56	−0.01	–0.70	-0.01	–0.71
EA_ps_					0.000005	2.93**	0.000001	2.94**
SES* EA_ps_							−0.00000006	–0.24
*R* ^2^		0.05		0.05		0.03		0.03
Intercept	2.91	116.33***	2.92	155.07***	3.30	34.53***	3.31	34.05***
SES			0.11	8.77***	0.10	10.85***	0.17	2.94**
Gender			−0.02	–1.56	−0.01	–0.74	−0.01	–0.75
STR					−0.03	−4.20***	−0.03	−4.17***
SES* STR							−0.01	–1.33
*R* ^2^		0.05		0.05		0.03		0.03
Intercept	2.91	116.33***	2.92	155.07***	2.89	167.24***		
SES			0.11	8.77***	0.09	10.44***		
Gender			−0.02	–1.56	−0.01	–0.74		
TMTA_pc_					0.08	1.81		
		0.05		0.05		0.03		

***p < 0.001, **p < 0.01, *p < 0.05.

We added provincial factors as level-2 predictor. PB_pc_ and STR significantly predicted behavioral engagement (β = 0.11, *t* = 2.72, *p* = 0.01, 95% *CI* = 0.03∼0.19, *R*^2^_PBpc_ = 0.91%; β = −0.04, *t* = −4.50, *p* = 0.00, 95% *CI* = −0.06∼−0.02, *R*^2^_STR_ = 0.91%), emotional engagement (β = 0.10, *t* = 2.24, *p* = 0.03, 95% *CI* = 0.01∼0.18, *R*^2^_PBpc_ = 1.27%; β = −0.03, *t* = −3.14, *p* = 0.00, 95% *CI* = −0.05∼−0.01, *R*^2^_STR_ = 1.28%), and cognitive engagement (β = 0.11, *t* = 3.53, *p* = 0.00, 95% *CI* = 0.05∼0.16, *R*^2^_PBpc_ = 0.70%; β = −0.03, *t* = −4.20, *p* = 0.00, 95% *CI* = −0.05 to 0.02). PHE and EA_ps_ significantly predicted behavioral engagement (β = 0.11, *t* = 3.04, *p* = 0.00, 95% *CI* = 0.04∼0.19, *R*^2^_PHE_ = 0.90%; β = 0.00000008, *t* = 2.88, *p* = 0.01, 95% *CI* = 0.00∼0.00, *R*^2^_EAps_ = 0.91%) and cognitive engagement (β = 0.09, *t* = 3.10, *p* = 0.00, 95% *CI* = 0.03∼0.16, *R*^2^_PHE_ = 0.70%; β = 0.000005, *t* = 2.93, *p* = 0.01, 95% *CI* = 0.00∼0.00, *R*^2^_EAps_ = 0.70%). GDP_pc_ significantly predicted cognitive engagement (β = 0.000001, *t* = 2.12, *p* = 0.04, 95% *CI* = 0.00∼0.00, *R*^2^_GDPpc_ = 0.70%). TMTA_pc_ could not predict behavioral, emotional, and cognitive engagement (β = 0.09, *t* = 1.71, *p* = 0.09; β = 0.04, *t* = 0.66, *p* = 0.51; β = 0.08, *t* = 1.81, *p* = 0.08). No interaction function was found. Detail information was in Appendix.

## Discussion

In this study, we used a multilevel model to test an ecological model of school engagement among adolescents from 31 provincial-level regions in China. Large-scale data were used to test a multilevel model predicting the school engagement of adolescents based on individual (gender), microsystem factors (SES), and provincial-level macrosystem factors (economy, public cultural facilities, technological industry, and education). The current study addressed important gaps in the literature concerning the effect of the macrosystem environment and the interaction between the microsystem and macrosystem on adolescents’ school engagement.

The current study overcame the deficit that a few studies concerned the relationship between adolescents and the macrosystem (Lam et al., 2015) and the interaction between the microsystem and macrosystem. This study examined the factors of microsystem factors and individual factors in relating to adolescents’ school engagement. Additionally, the study examined provincial-level macrosystems that may differentially relate to adolescents’ engagement in school.

Consistent with prior studies, no gender difference was found in adolescents’ school engagement. According to Bronfenbrenner’s theory, processes, including the interaction of individuals and context, affect adolescents’ development. Sex differences in the way of interaction might cause the same development outcome. Girls perceived more social support than boys (Rueger et al., 2009). Social support was a significant predictor of school engagement (Wang and Eccles, 2012b). Boys’ engagement was significantly predicted by grade, while girls’ engagement was significantly predicted by anxiety classification (Wilcox et al., 2017). However, there was no significant gender difference in school engagement (Janosz et al., 2008; Steinmayr and Spinath, 2008; Zendarski et al., 2017).

Consistent with prior studies, the microsystem factor (SES) positively predicted school engagement. According to Bronfenbrenner’s (1989) theory, as a microsystem factor, the family environment is the most proximal context and continually influences adolescents’ development. Adolescents from low-SES families always face more stresses and challenges, such as fewer educational resources and low parental involvement (Duan et al., 2018). Educational resources and parental involvement were positive predictors of engagement (Xiong et al., 2021).

The influence of provincial-level factors on school engagement was examined. The bioecological model suggested that adolescents were influenced by unrecognized macrosystems. The current study provided evidence that the macrosystem environment influenced behavioral, emotional, and cognitive engagement in different ways. They were all influenced by the PB_pc_ and STR. GDP_pc_ only predicted cognitive engagement but not behavioral engagement and emotional engagement. PHA and EA_ps_ predicted behavioral engagement and cognitional engagement, but not emotional engagement. The results revealed the interaction between adolescents and the macrosystem.

Conceptual frameworks explaining the effects of economic development on adolescents’ development include three potential pathways. First, economic growth and recession affect parents’ job stability and income, which in turn affects the development of children and adolescents. In the United States, during economic depressions, children show more academic disorders and behavioral problems (Weiland and Yoshikawa, 2012). Second, education funding was unlikely to be cut back on in a rich region. An investigation of education development (educational opportunities, educational facilities, teacher resources and educational output) of 31 provincial regions in China found that regions with flourishing economies had higher levels of educational development (Wang et al., 2013). Third, Adolescents living in low-income area were at higher risk for development. Low-income area and high-income area were different in institutional resources, such as grocery stores with healthy food, out-of-school programs, public services and transportation (Huston and Bentley, 2010). Parenting warmth and discipline was influenced by the stresses of living in a low-income neighborhood (Pinderhughes et al., 2001).

The improvement of the public cultural service system was conducive to school engagement for adolescents. Public cultural services include non-profit public cultural products and services, such as libraries and museums. Public cultural services provide various and rich learning resources for adolescents, such as different kinds of books and historical relics. No influence of scientific and technological industry development on school engagement was found. Science and technology institutions are the end users and consumers of the education system and do not participate in the education process in which adolescents acquire knowledge and skills (Kapitzke and Hay, 2011). Therefore, there was no connection between technology and what adolescents were learning, and it could not improve their awareness of knowledge instrumentality. Gratifyingly, education investment significantly predicted adolescents’ school engagement. Educational appropriations were an indispensable financial condition for improving school hardware facilities and improving the number of teachers. Teachers were the primary interlocutors for adolescents in school. Their importance is self-evident. The education degree of residents was the way for adolescents to realize the results of education. A higher education degree was always accompanied by higher social status and income (Bradley and Corwyn, 2002). It provided an external stimulus for adolescents engaging in school activities.

There was little difference between the microsystem environment (SES) and the macrosystem environment (provincial factors) on the effect of adolescents’ school engagement. This revealed that the macrosystem environment influenced adolescents’ school engagement in an indirect and powerful way. Macrosystem factors should be considered in future studies about adolescents’ school engagement. This study also provided potential policies for the government to enhance students’ academic development, including increasing the volume of books in public libraries and setting up more teachers’ positions.

There were three limitations in this study. First, the variations in the sample size across provincial-level regions were great, and the grades of sampled schools were not balanced. These factors might lead to errors caused by sampling bias. Second, the use of a cross-sectional research design in the present study limited any causal conclusions about the findings and does not allow for “change over time” effects to be examined. A repeated measured longitudinal study would be useful for examining time effects on school engagement. Third, the province-level indicators in this study reflected the average development level of the whole province, which vaguely reflects the regional environment of the subjects. This might be why this study failed to find an interaction between the provincial environment and SES. A smaller range of regional development indicators (such as city and town) could more accurately reflect the characteristics of the environment. In future studies, selecting a smaller range of regional development indicators would be helpful in exploring the effect of the interaction between the regional environment and family environment on school engagement. Fourth, there were several items about adolescents’ feelings of teachers and classes in the questionnaire. When conducting the survey, there was no special provision for teachers and classrooms. That cause they might be thinking about different teachers and classes when the adolescents answer the questionnaire.

## Conclusion

This study had important implications for understanding adolescents’ perceptions of their microsystem and school engagement within and between macrosystems. The ecological model of adolescents was supported in the current study. The ecological model provided a potential framework for future studies assessing adolescents’ school engagement in a variety of environments. The results revealed the possibility of using ecological models to understand adolescent development.

## Data Availability Statement

The raw data supporting the conclusions of this article will be made available by the authors, without undue reservation.

## Author Contributions

FL contributed to the research design, data collection and analyses, and writing. XG provided useful suggestions in the research design stage. LX contributed to the data analyze. XW contributed to editing the manuscript. HW contributed to organizing the database. All authors contributed to the article and approved the submitted version.

## Conflict of Interest

The authors declare that the research was conducted in the absence of any commercial or financial relationships that could be construed as a potential conflict of interest.

## Publisher’s Note

All claims expressed in this article are solely those of the authors and do not necessarily represent those of their affiliated organizations, or those of the publisher, the editors and the reviewers. Any product that may be evaluated in this article, or claim that may be made by its manufacturer, is not guaranteed or endorsed by the publisher.

## Footnotes

1 Liu, F., Gai, X., Wu, X., and Wang, H. (Submitted). National norm and status quo of school engagement in middle school and college.
