# Supplementary material for: School Engagement and Context: A Multilevel Analysis of Adolescents in 31 Provincial-Level Regions in China
Source: Front Psychol. 2021 Oct 26;12:724819. doi: 10.3389/fpsyg.2021.724819 (PMC8576515; doi:10.3389/fpsyg.2021.724819)
Supplement: Supplementary file 1 [file Table_1.DOCX]

Table 1

Sample distribution in 31 provincial regions

| Provincial Region | *N* | Gender | | Grade | | |
| --- | --- | --- | --- | --- | --- | --- |
|  |  | Male | Female | 7_th_ | 8_th_ | 9_th_ |
| Beijing | 590 | 275 | 315 | 183 | 309 | 98 |
| Tianjin | 87 | 44 | 43 | 73 | 14 | 0 |
| Hebei | 88 | 52 | 36 | 29 | 38 | 20 |
| Shanxi | 199 | 87 | 112 | 128 | 38 | 32 |
| Inner Mongolia | 650 | 318 | 332 | 422 | 223 | 5 |
| Liaoning | 1645 | 847 | 798 | 872 | 736 | 36 |
| Jilin | 4957 | 2421 | 2536 | 2475 | 2182 | 294 |
| Heilongjiang | 679 | 370 | 309 | 5 | 363 | 310 |
| Shanghai | 239 | 108 | 131 | 126 | 113 | 0 |
| Jiangsu | 476 | 188 | 287 | 367 | 107 | 1 |
| Zhejiang | 512 | 249 | 256 | 166 | 182 | 164 |
| Anhui | 397 | 225 | 172 | 184 | 117 | 93 |
| Fujian | 448 | 198 | 250 | 262 | 186 | 0 |
| Jiangxi | 150 | 64 | 36 | 45 | 51 | 3 |
| Shandong | 95 | 41 | 54 | 73 | 20 | 0 |
| Henan | 160 | 82 | 78 | 3 | 120 | 37 |
| Hubei | 862 | 552 | 306 | 380 | 352 | 130 |
| Hunan | 94 | 40 | 54 | 2 | 91 | 1 |
| Guangdong | 3490 | 1900 | 1590 | 2153 | 1314 | 14 |
| Guangxi | 397 | 220 | 177 | 203 | 192 | 2 |
| Hainan | 58 | 31 | 27 | 56 | 2 | 0 |
| Chongqing | 130 | 58 | 72 | 41 | 76 | 12 |
| Sichuan | 582 | 275 | 301 | 182 | 194 | 206 |
| Guizhou | 160 | 70 | 89 | 98 | 61 | 0 |
| Yunnan | 995 | 453 | 542 | 751 | 210 | 34 |
| Tibet | 42 | 21 | 21 | 0 | 40 | 0 |
| Shaanxi | 110 | 59 | 51 | 33 | 23 | 54 |
| Gansu | 116 | 62 | 53 | 42 | 74 | 0 |
| Qinghai | 238 | 96 | 142 | 73 | 80 | 85 |
| Ningxia | 272 | 135 | 137 | 189 | 73 | 10 |
| Xinjiang | 166 | 52 | 114 | 20 | 61 | 82 |
| Total | 19084 | 9593 | 9421 | 9636 | 7642 | 1723 |

Table 2

Mean and standard deviation of school engagement of different provincial regions

| Provincial Region | Mean | Std. Deviation |
| --- | --- | --- |
| Beijing | 3.21 | 0.48 |
| Tianjin | 3.06 | 0.52 |
| Hebei | 2.81 | 0.42 |
| Shanxi | 3.01 | 0.53 |
| Inner Mongolia | 2.97 | 0.57 |
| Liaoning | 3.14 | 0.55 |
| Jilin | 3.05 | 0.58 |
| Heilongjiang | 3.06 | 0.57 |
| Shanghai | 3.23 | 0.52 |
| Jiangsu | 3.07 | 0.51 |
| Zhejiang | 2.86 | 0.43 |
| Anhui | 3.09 | 0.48 |
| Fujian | 2.93 | 0.52 |
| Jiangxi | 2.64 | 0.50 |
| Shandong | 3.13 | 0.50 |
| Henan | 3.08 | 0.51 |
| Hubei | 2.87 | 0.53 |
| Hunan | 2.73 | 0.46 |
| Guangdong | 2.95 | 0.50 |
| Guangxi | 2.97 | 0.47 |
| Hainan | 2.99 | 0.45 |
| Chongqing | 3.05 | 0.45 |
| Sichuan | 2.97 | 0.47 |
| Guizhou | 2.76 | 0.44 |
| Yunnan | 2.85 | 0.48 |
| Tibet | 3.01 | 0.48 |
| Shaanxi | 2.83 | 0.51 |
| Gansu | 2.82 | 0.55 |
| Qinghai | 2.86 | 0.44 |
| Ningxia | 3.24 | 0.51 |
| Xinjiang | 3.07 | 0.48 |

Table3.

Correlation of Provincial indicators.

|  | 1 | 2 | 3 | 4 | 5 | 6 |
| --- | --- | --- | --- | --- | --- | --- |
| GDP_pc_ | 1 |  |  |  |  |  |
| PB_pc_ | .78^**^ | 1 |  |  |  |  |
| PHE | .71^**^ | .58^**^ | 1 |  |  |  |
| EA_ps_ | .77^**^ | .65^**^ | .84^**^ | 1 |  |  |
| STR | -.35 | -.38^*^ | -.64^**^ | -.60^**^ | 1 |  |
| TMTA_pc_ | .68^**^ | .39^*^ | .89^**^ | .85^**^ | -.53^**^ | 1 |

^**^ *p*<.01, ^*^ *p*<.05

Table 4.

School engagement questionnaire

Please choose the description that fits you. ①= strongly disagree; ②= disagree; ③= agree; ④= strongly agree.

|  | Item | ① | ② | ③ | ④ |
| --- | --- | --- | --- | --- | --- |
| 1 | When I read a book, I ask myself questions to make sure I understand what's in the book. (Cognitive engagement) |  |  |  |  |
| 2 | I feel boring in class. (Emotional Engagement) |  |  |  |  |
| 3 | I work hard at school. (Behavioral Engagement) |  |  |  |  |
| 4 | I always check my homework. (Cognitive engagement) |  |  |  |  |
| 5 | I always look forward to the end of class. (Emotional Engagement) |  |  |  |  |
| 6 | If I meet something I do not understand while reading, I will try to understand it by asking others or looking it up in the dictionary. (Cognitive engagement) |  |  |  |  |
| 7 | I take an active part in class discussions. (Behavioral Engagement) |  |  |  |  |
| 8 | I do not like studying. (Emotional Engagement) |  |  |  |  |
| 9 | If I find something difficult to understand, I will read it again and again. (Cognitive engagement) |  |  |  |  |
| 10 | I always feel sleepy in class. (Emotional Engagement) |  |  |  |  |
| 11 | I pay attention in class. (Behavioral Engagement) |  |  |  |  |
| 12 | I relate new knowledge to my own experience. (Cognitive engagement) |  |  |  |  |
| 13 | I study carefully in class. (Behavioral Engagement) |  |  |  |  |
| 14 | I like my school. (Cognitive engagement) |  |  |  |  |
| 15 | I think deeply about the material, not just skim it. (Emotional Engagement) |  |  |  |  |
| 16 | Class is very interesting. (Emotional Engagement) |  |  |  |  |
| 17 | I pretend to be studying in class. (Behavioral Engagement) |  |  |  |  |
| 18 | I try to relate new knowledge to other subjects. (Cognitive engagement) |  |  |  |  |

*Note.* Item 8,9,10,11,18 is reverse-scored.
